# Supplementary material for: Metastatic adrenocortical carcinoma displays higher mutation rate and tumor heterogeneity than primary tumors
Source: Nat Commun. 2018 Oct 9;9:4172. doi: 10.1038/s41467-018-06366-z (PMC6178360; doi:10.1038/s41467-018-06366-z)
Supplement: Supplementary file 1 — Supplementary Information [file 41467_2018_6366_MOESM1_ESM.pdf]

Metastatic adrenocortical carcinoma displays higher mutation rate and tumor heterogeneity than primary tumors

Gara et al.

Supplementary Information

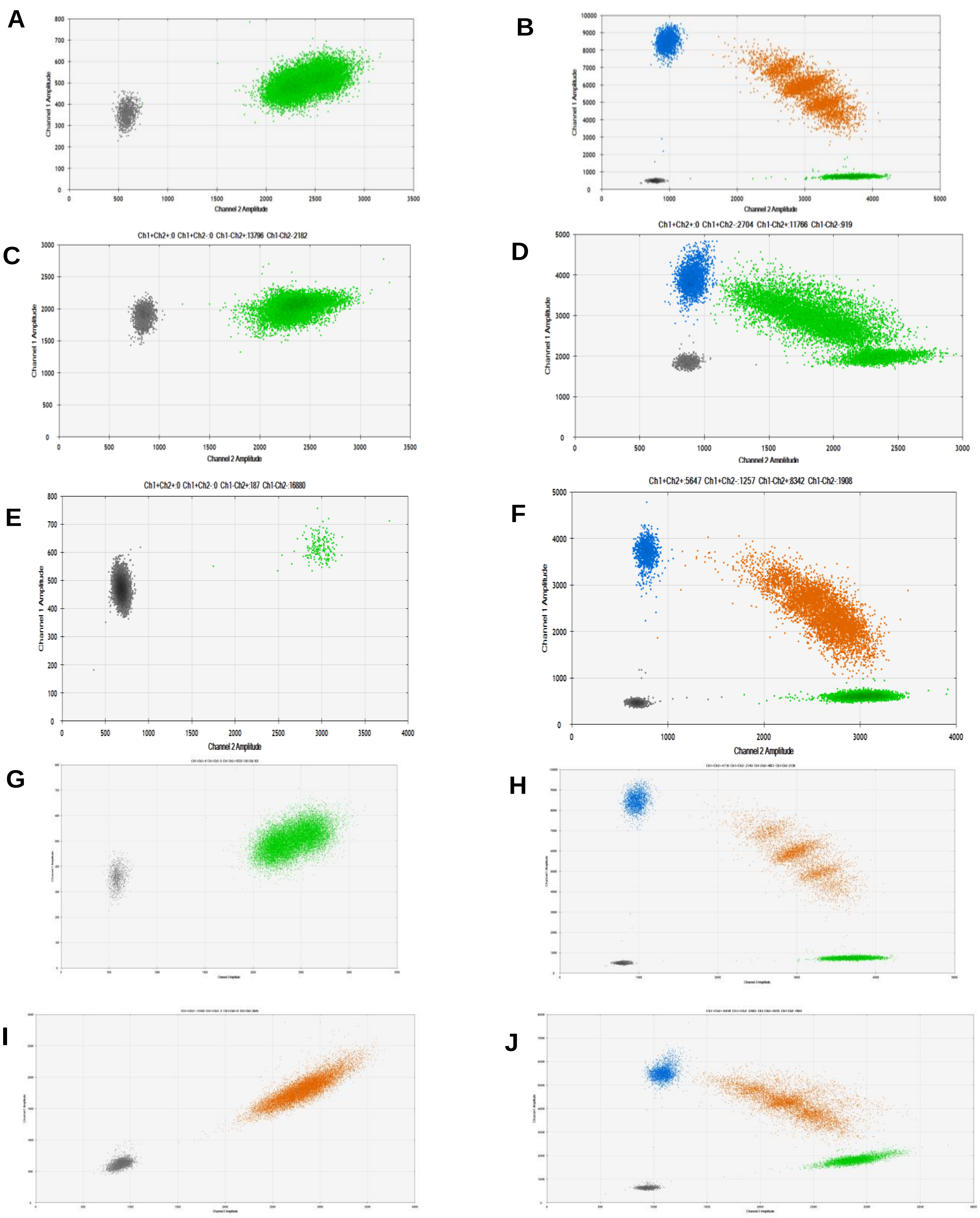

Supplementary Figure 1

Validation of genetic variants that was identified in our study through droplet-digital PCR. The scatter plots represent germline (A,C, E G and I) and tumor samples (B, D, F, H and J) from each case. The selected variants are *CTNNB1*_pG34R (A&B), *CTNNB1*_pD32G (C&D), *DNHD1*_pE2985K (E&F), *WD66*_pA928S (G&H) and *ENTHD1*_pN196T (I&J)

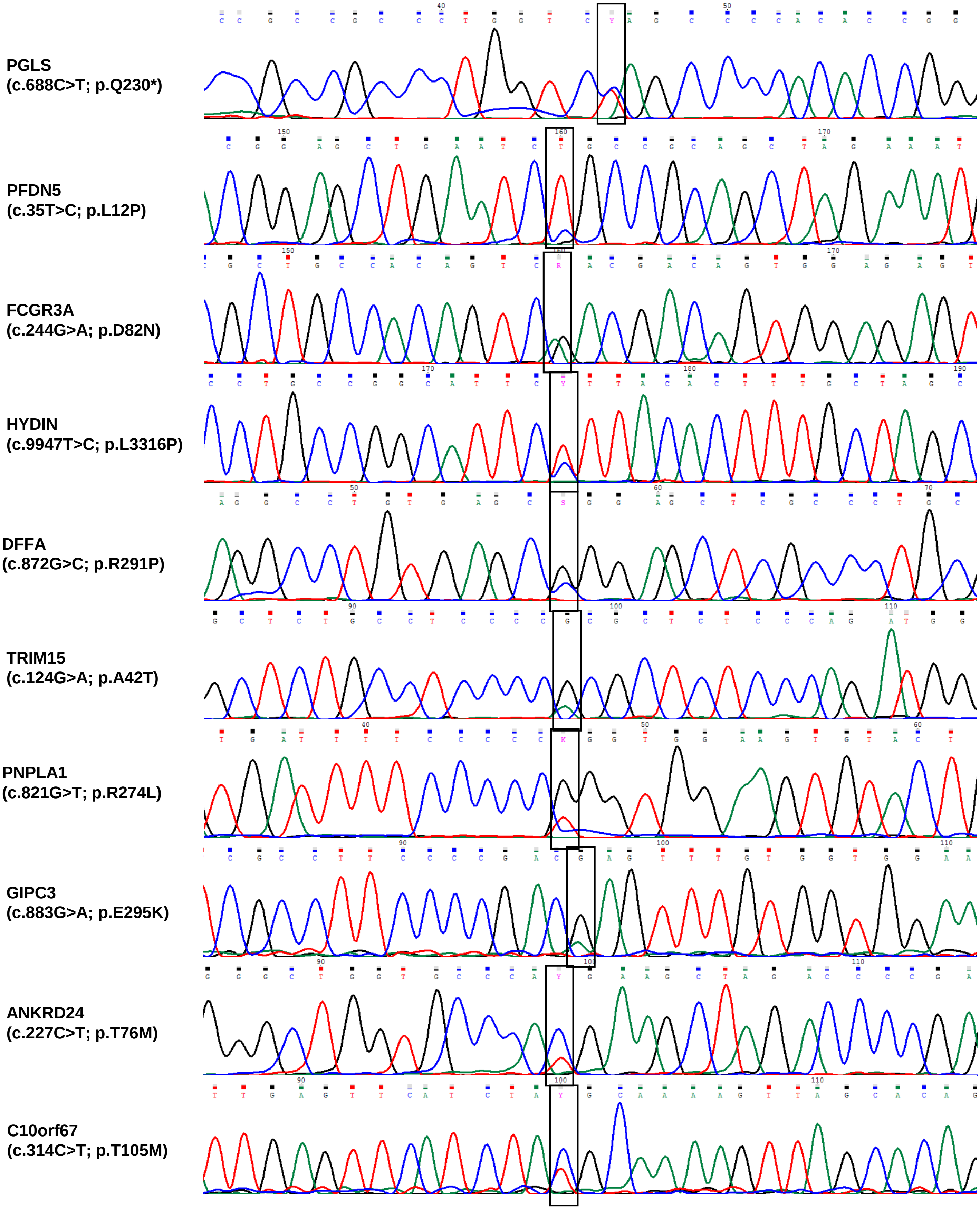

Supplementary Figure 2: Validation of genetic variants that was identified in our study through Sanger sequencing. The DNA sequence chromatogram profile of 10 randomly selected genetic variants in different tumors. The cDNA and amino acid change was on the left side of the chromatogram (boxed region in the chromatogram is the location of the mutation)

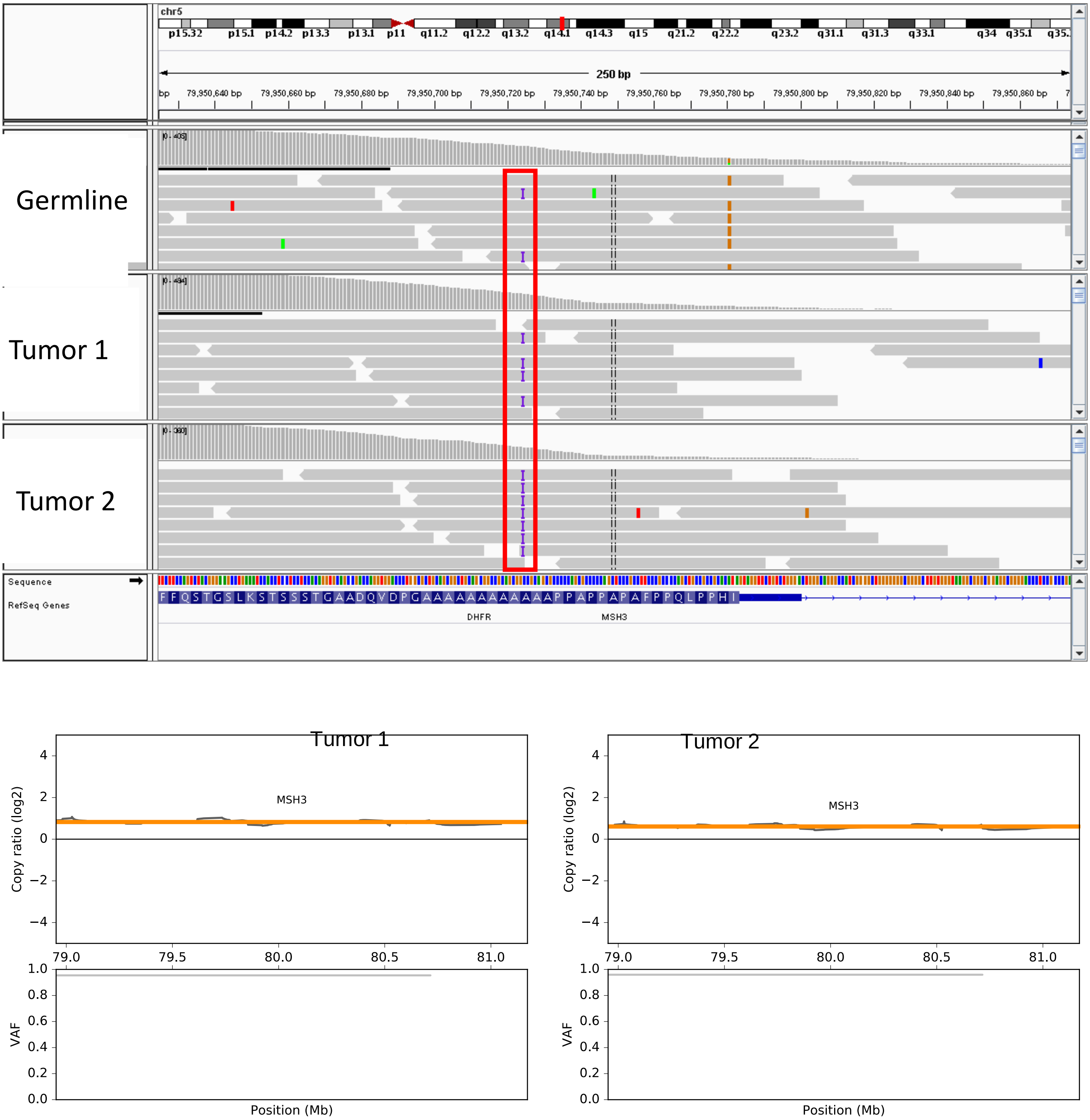

Supplementary Figure 3. *MSH3* loss of function germline mutation in hypervariable ACC metastases tumor 1 and 2. The to panel is an IGV BAM file image illustrating the 9-base INDEL in the germline and two metastases from a single patient. The lower panels show log2 ratios and b-allele frequencies generated with CNVkit. log2 ratios show copy number gain at this locus, and b-allele frequencies show the total loss of heterozygosity, fixing this mutation in both tumor samples.

A.

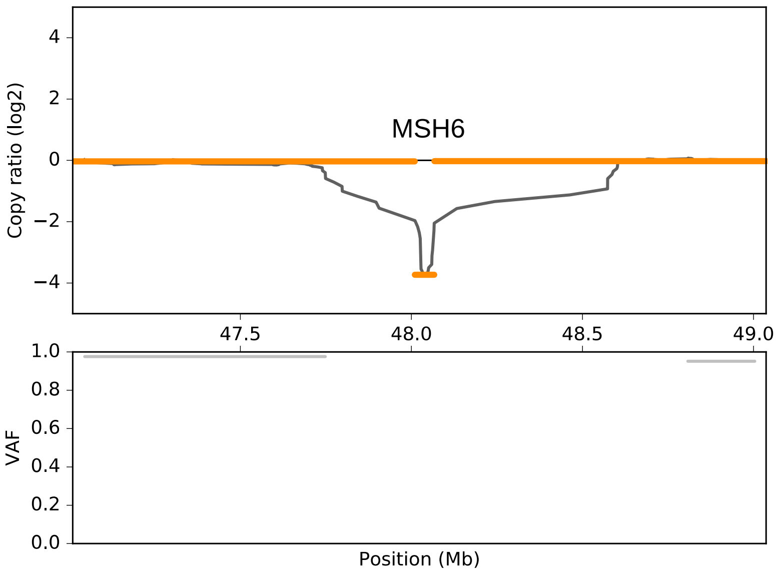

B.

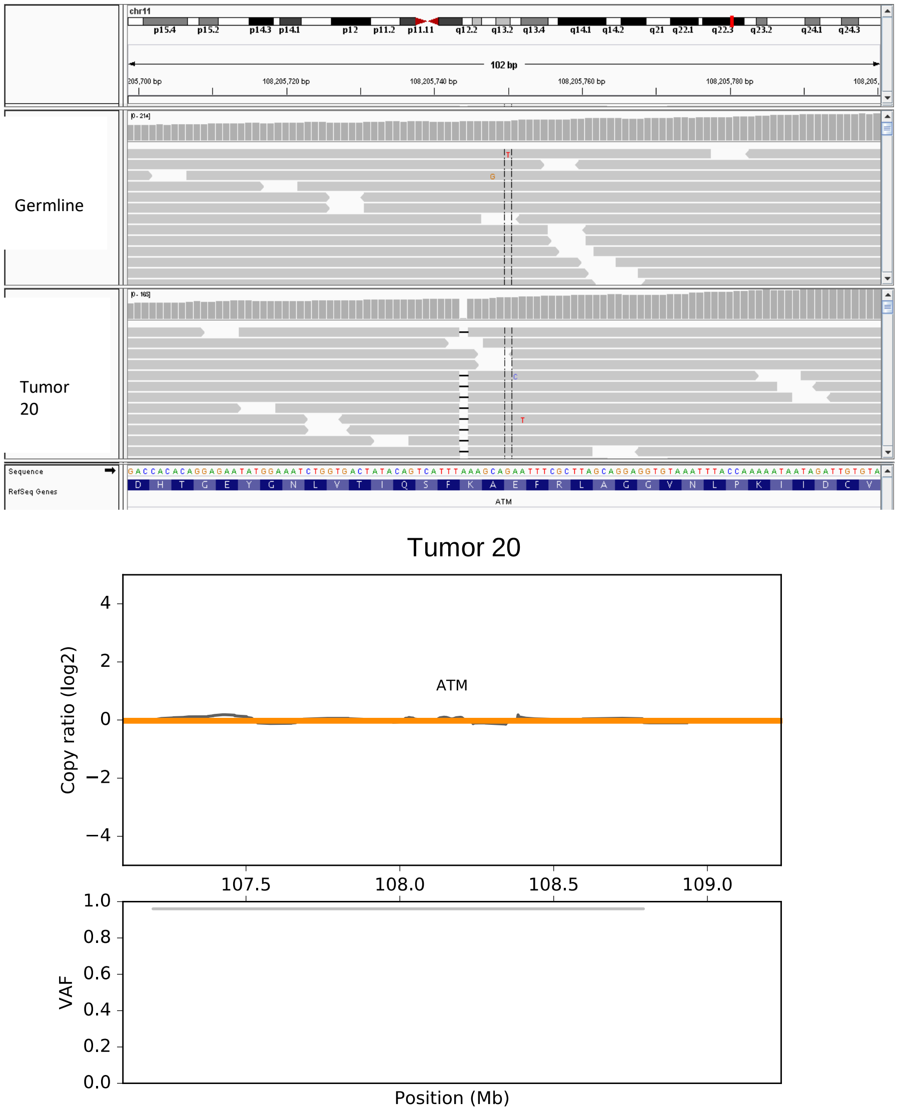

Supplementary Figure 4. *MSH6* homozygous deletion (A) and *ATM* frameshift (B) in hypervariable ACC metastasis tumor 20.

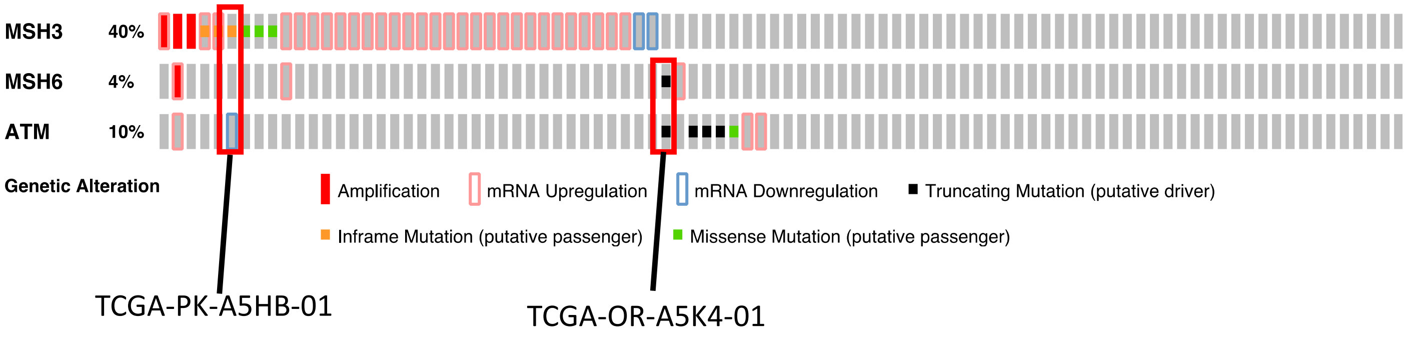

Supplementary Figure 5. Frequency of somatic mutations in 92 primary ACCs available in cBioportal for the three genes putatively driving hypermutation in ACC metastases.

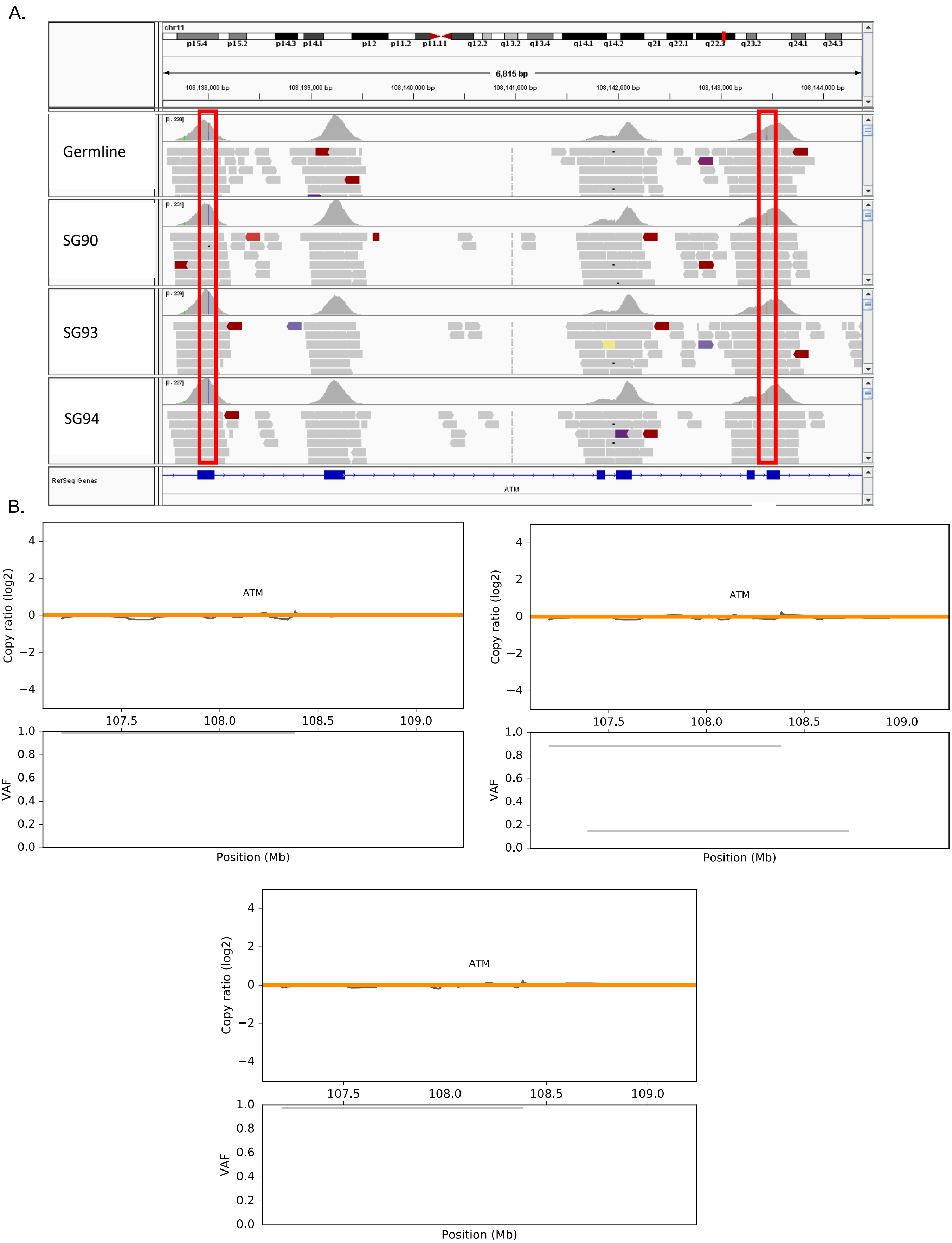

Supplementary Figure 6. *ATM* missense mutations in (A) and *ATM* frameshift (B) in hypervariable ACC metastasis tumor 20.

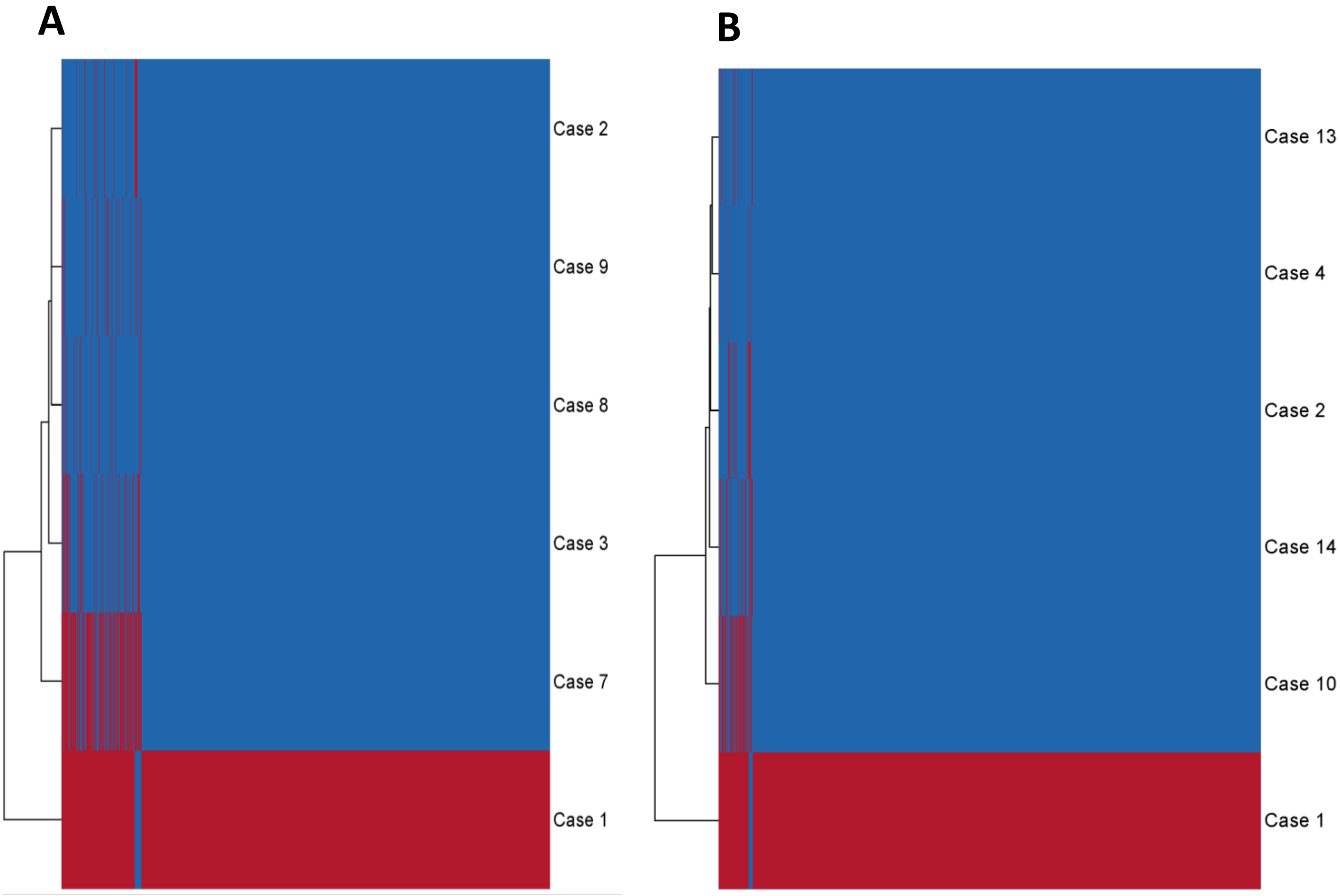

Supplementary Figure 7. Heatmaps and clustering of overlapping variants within the genes in metastatic lung (A) and other tumor sites (B) together with the hypermutator case

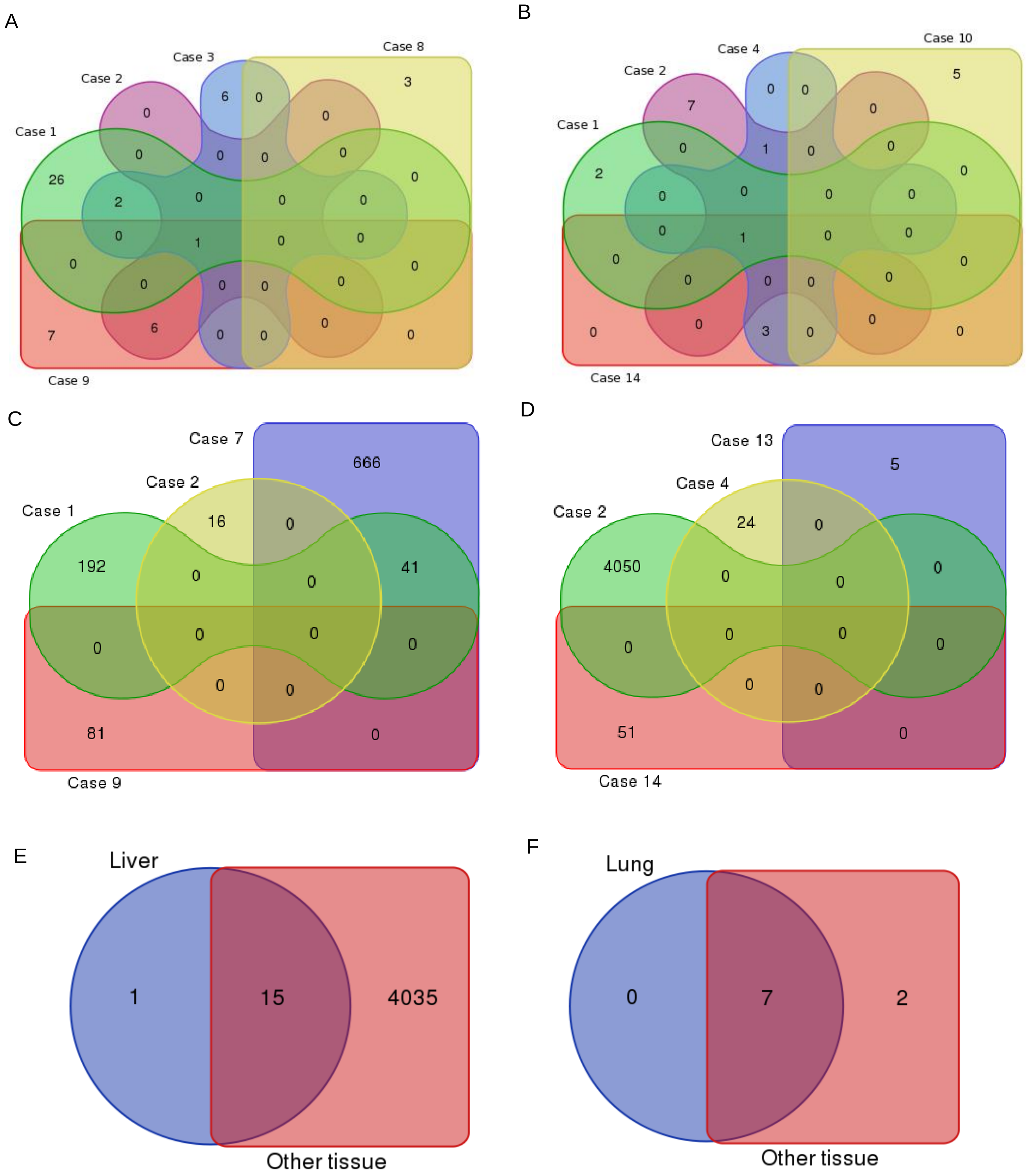

Supplementary Figure 8

Copy number variation in metastatic ACC tumors. Venn diagrams representing the total number of homozygous deletions and high level amplifications in four different metastatic lung (A & C) and other tissue tumors (B &D) respectively. The homozygous deletions (E) and amplifications (F) in a case with two metastatic sites; lung and other tissues.

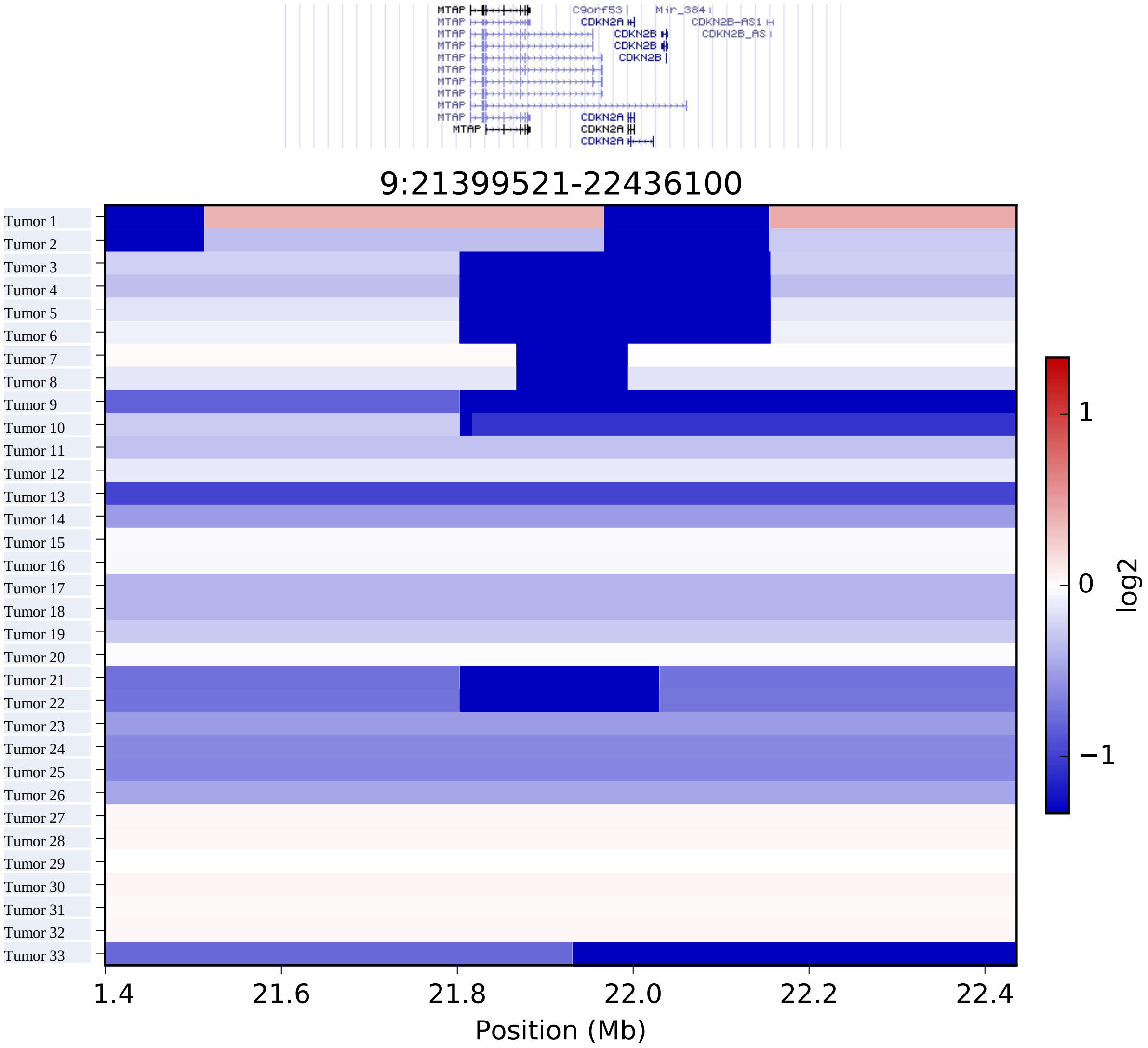

Supplementary Figure 9. Heatmap illustration of recurrent *CDKN2A/CDKN2B/MTAP* deletion

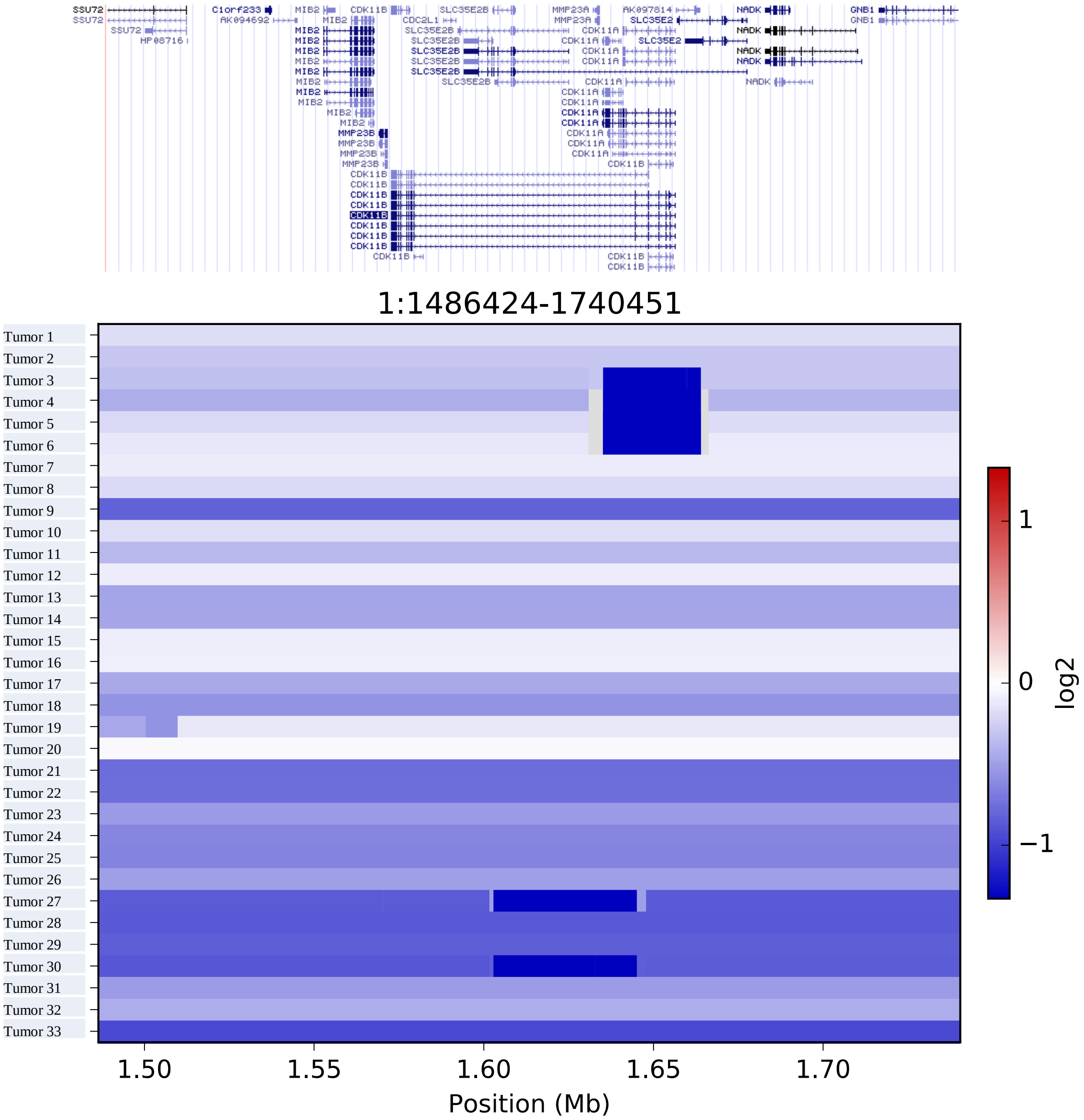

Supplementary Figure 10. Heatmap illustration of recurrent *CDK11A/CDK11B* deletion

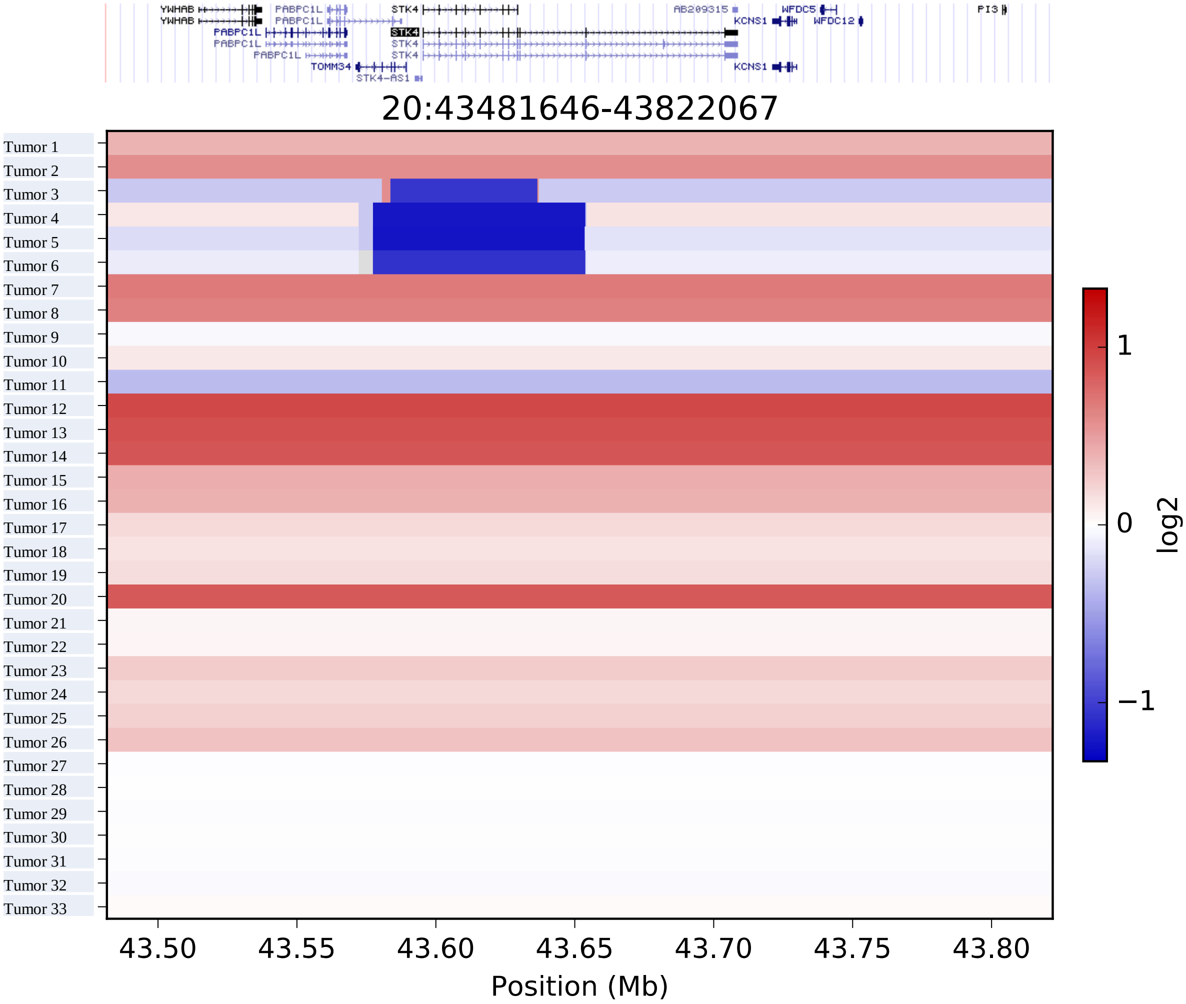

Supplementary Figure 11. Heatmap illustration of recurrent *STK4/TOMM34* deletion

Cases	Sample	Tumor Purity	Subclonal Genome Fraction	SNP Counts All SNPs	Coding Nonsilent	Coding Silent	SNPs PerMegabase All SNPs	Coding Nonsilent	Coding Silent	MuTect Covered Bases	Proportion CNV altered	Proportion LOH
Case 1	Tumor 1	0.92	0.0100	6551	2494	4057	140.8979	53.6406	87.2573	46494669	0.4007	0.7930
Case 1	Tumor 2	1.00	0.0000	3667	1428	2239	80.6711	31.4149	49.2562	45456191	0.9382	0.7243
Case 2	Tumor 3	0.85	0.0200	98	34	64	2.2390	0.7768	1.4622	43770135	0.7221	0.5048
Case 2	Tumor 4	0.86	0.0100	92	35	57	2.1301	0.8104	1.3197	43190006	0.6736	0.5447
Case 2	Tumor 5	0.94	0.0000	121	38	83	2.7809	0.8733	1.9076	43510708	0.5102	0.4739
Case 2	Tumor 6	0.81	0.0100	116	41	75	2.6278	0.9288	1.6990	44143875	0.4345	0.4110
Case 3	Tumor 7	1.00	0.0000	159	63	96	3.5203	1.3948	2.1255	45166882	0.3682	0.6762
Case 3	Tumor 8	0.75	0.0100	217	93	124	4.7627	2.0412	2.7215	45562460	0.3931	0.7054
Case 4	Tumor 9	1.00	0.0000	67	24	43	1.5793	0.5657	1.0136	42424559	0.2907	0.1974
Case 5	Tumor 10	0.31	0.0100	215	91	124	4.9358	2.0891	2.8467	43559540	0.3096	0.1629
Case 6	Tumor 11	1.00	0.0100	147	65	82	3.2296	1.4281	1.8016	45515892	0.4950	0.4108
Case 7	Tumor 12	0.85	0.0100	504	224	280	11.2288	4.9906	6.2382	44884404	0.4627	0.2320
Case 7	Tumor 13	0.90	0.0200	450	185	265	10.1537	4.1743	5.9794	44318620	0.6178	0.3827
Case 7	Tumor 14	0.96	0.0100	537	207	330	12.1514	4.6840	7.4673	44192542	0.5157	0.3144
Case 8	Tumor 15	1.00	0.0000	114	42	72	2.5841	0.9520	1.6320	44116768	0.2833	0.8075
Case 8	Tumor 16	0.93	0.0000	118	40	78	2.6376	0.8941	1.7435	44737744	0.2973	0.8082
Case 9	Tumor 17	1.00	0.0100	126	53	73	2.7770	1.1681	1.6089	45372897	0.4082	0.2999
Case 9	Tumor 18	0.92	0.0100	147	60	87	3.2758	1.3371	1.9388	44873924	0.3667	0.2583
Case 9	Tumor 19	1.00	0.0000	144	60	84	3.1843	1.3268	1.8575	45222271	0.4248	0.2650
Case 10	Tumor 20	0.93	0.0100	574	259	315	12.3841	5.5880	6.7962	46349581	0.1669	0.8387
Case 11	Tumor 21	0.73	0.0000	66	30	36	1.5174	0.6897	0.8277	43495518	0.5106	0.5091
Case 11	Tumor 22	0.84	0.0000	86	32	54	1.9700	0.7330	1.2370	43655497	0.5106	0.5163
Case 12	Tumor 23	0.97	0.0200	77	27	50	1.7303	0.6067	1.1236	44499729	0.9597	0.4837
Case 12	Tumor 24	0.92	0.0100	81	26	55	1.8852	0.6051	1.2801	42966871	0.9437	0.4996
Case 12	Tumor 25	0.83	0.0100	72	23	49	1.7166	0.5483	1.1682	41944315	0.9024	0.4981
Case 12	Tumor 26	0.87	0.0100	83	32	51	1.8567	0.7158	1.1408	44703883	0.9585	0.4884
Case 13	Tumor 27	0.89	0.0000	79	24	55	1.7775	0.5400	1.2375	44443520	0.5231	0.5472
Case 13	Tumor 28	0.89	0.0100	94	28	66	2.1214	0.6319	1.4895	44310782	0.5256	0.5356
Case 13	Tumor 29	0.83	0.0100	87	27	60	1.9420	0.6027	1.3393	44798925	0.5252	0.5473
Case 13	Tumor 30	0.88	0.0100	84	25	59	1.8631	0.5545	1.3086	45086784	0.5251	0.5456
Case 13	Tumor 31	0.38	0.0000	89	26	63	2.0584	0.6013	1.4571	43237553	0.5172	0.5461
Case 13	Tumor 32	0.83	0.0000	75	21	54	1.6964	0.4750	1.2214	44212491	0.5130	0.4979
Case 14	Tumor 33	0.78	0.0000	184	71	113	4.0390	1.5585	2.4805	45555279	0.4199	0.3524
	Mean			464.27	179.64	284.64	10.18	3.94	6.24		0.5277	0.4963
	Median			116	40	73	2.63	0.89	1.63		0.5106	0.4996

Supplementary Table 1

Mutational summary of all ACC metastatic tumors. Tumor purity and subclonal genome fraction generated in ABSOLUTE. SNP count and frequency data generated using MuTect. 'All SNPs' refers to exonic (including synonymous), UTR, and splicing mutations; 'Coding Nonsilent' refers to exonic mutations excluding synonymous mutations and splice mutations; 'Coding Silent' refers to synonymous, UTR, and splice mutations. Tumors 1&2 exhibit hyper mutation phenotype belong to the same patient.

Gene Symbol	Chr	Start Position (hg19)	Stop Position (hg19)	Mutation/Event	Ref Allele	Tumor Allele	Mutation Location	Samples Positive
ATM	11	108138003	108138003	Missense Mutation	T	C	Germline, with Somatic LoH	Tumors 12, 13 &14
ATM	11	108143456	108143456	Missense Mutation	C	G	Germline, with Somatic LoH	Tumors 12, 13 &14
ATM	11	108205744	108205744	FrameShift Deletion	A	-	Somatic, with LoH	Tumor 20
MSH3	5	79950724	79950724	InFrame Insertion	G	GCCGCAGCGC	Germline, with Somatic LoH	Tumors 1&2
MSH6	2	48010242	48036835	HomozygousDeletion	NA	NA	Somatic	Tumor 20

Supplementary Table 2

Putative mutational events underlying hypermutation phenotypes in ACC metastases. LoH = Loss of Heterozygosity

Cases	Total genes	Gene name/s
Case 1 Case 2 Case 7	1	DNAH6
Case 1 Case 3 Case 7	2	MUC5B HELZ2
Case 1 Case 7 Case 8	1	KIAA0100
Case 1 Case 2	7	ZNF646 FHDC1 VSTM2B SNRNP200 TAS2R41 CKAP5 RASGRF2
Case 1 Case 3	21	SALL1 MYO9B ARHGAP21 TENM4 CLEC1B TTLL4 CAMSAP1 LRP1B DCDC1 KCNH3 PFAS ZNF44 CHD7 DMXL2 FSIP2 ITGAX MTFMT SHANK1 CACNA1F MAP2 CFTR
Case 1 Case 7	43	PLCE1 TET3 SUSD2 AHI1 MEGF10 PIEZO2 COL5A2 FAM120A MKLN1 CD93 KCNN3 CDH11 IKBKAP CCT7 TTC40 TULP1 CNTN1 BRD7 RBP3 MUM1 DRD1 MUC3A PPP6R1 MYH3 ANK2 VWF NEB ZFR2 RNF213 BAG6 TRIM67 PTPRD CACNA1A KCNQ2 TROVE2 IGF1R BCAS1 SALL4 CCDC135 TTN NRCAM COL6A3 FAM65C
Case 1 Case 8	8	FN1 CWH43 DNAH7 NAV3 ANKS1B OBSCN RBL2 ADRA2B
Case 1 Case 9	10	DNAH11 DNHD1 ACTN1 APC TMTC1 KIAA1549L SAMD9L ADAMTS20 SNTB2 MMP9
Case 2 Case 7	2	CPNE4 CTNNB1
Case 2 Case 9	1	EME1
Case 3 Case 8	1	MN1

Supplementary Table 3
The list of genes that are frequently mutated in metastatic adrenal tumors from lung.

Cases	Total genes	Gene name
Case 1 Case 10 Case 4	1	ARHGEF28
Case 1 Case 10 Case 14	1	PPL
Case 1 Case 2	1	CACNA2D4
Case 1 Case 4	2	MUM1L1 ARID1B
Case 1 Case 10	37	DCHS2 TRIB3 CHD9 FRY DIP2A CCDC141 TMEM63C PLEKHG4 LILRA6 PCLO GRIP1 ANK2 CREBBP HELZ2 SHANK1 TNFRSF1A PIEZO1 NLRX1 SPEG PHRF1 SRCAP KMT2D NCKAP5 MRGPRG DIP2B TTC28 PALM3 FGD1 UNC80 TTC17 HIVEP3 DMWD WDR52 RELN ANO6 COQ2 CDHR2
Case 1 Case 13	6	INF2 VWF KLHL8 ABCA4 TTN SCAF11
Case 1 Case 14	13	MYO9B VANGL2 SMCHD1 EOMES DUOX1 PCDH12 ABL1 SYCP2 SHANK2 NHSL1 DDX60L DDR2 PCIF1
Case 10 Case 2	2	CNOT1 DST
Case 14 Case 2	2	LILRA4 CTNNB1
Case 10 Case 4	1	PDE4DIP
Case 10 Case 14	1	GAL3ST3
Case 13 Case 14	2	PDZRN4 NOVA1

Supplementary Table 4
The list of genes that are frequently mutated in metastatic adrenal tumors from other tissue.

S.No	Primer sequence	Direction	Gene
1	CGGATCTGTGCACAGGACTGC	FP	TUBA3D
2	GAGTTGTAGGGCTCCACCACGG	RP	
3	CCACTTCTCTGTGTTTAACAG	FP	LTF
4	AAGGGGACAGGGTCACTCA	RP	
5	CACCCAGTGCCTCATGCAGCATG	FP	FAM205A
6	ATCGCAAGCCCCGTTGGCCTCT	RP	
7	TCGCAGATATCAAGGCCCG	FP	ASXL3
8	CAACCGGGTCTCTTTAGAGGTCT	RP	
9	CATCCGCAATCCTGTCAGCTTG	FP	ADAMTS4
10	TGCGGTGCTGACTGGGGCCT	RP	
11	GTCACCTTCCTCTCTTGCCC	FP	ITGA9
12	CAATCTCTCCTGGAACGTGTTTG	RP	
13	CTGGGCTTGTCTCCCGTGTG	FP	FRS3
14	TGCGTGCTCACCTGCTCATCAGGA	RP	
15	CTTTGTCCCTGCATCCAG	FP	DUSP5
16	GAAGGGGAATGGCTAAACCTAC	RP	
17	CGGCACAATAAAGCCACGGAAAAC	FP	GPR65
18	CATATCTTCCTGTTTCGGTTAC	RP	
19	CACCAAGCTACCCGAACTATG	FP	ZFR2
20	CATGGCAGGAGGGCGACTGAC	RP	
21	GCAATGGACAGAATACACTTAG	FP	ZNF614
22	TGAGTCCTCTGATGCTTGAAGAC	RP	
23	GAGTATGAAGCCTTGGAGTGGC	FP	FAM205A
24	GATGCTCACGTTGGCTAGGGCTGTG	RP	
25	GGCCCTTTTACTCTTTCATGCC	FP	CLIC5
26	CCTGTGGGTAGAGCTCTCTTAC	RP	
27	ATCCATCCCATCCTACTGCCAG	FP	PER3
28	CTGATGCTGCTGAACCAGTTCTGG	RP	
29	AGTACGGCGGTCACCTGGTGT	FP	IQSEC3
30	TCGGTGAAGGAGTCCTCCAGC	RP	
31	ATGGCTCTGGCGGTGGCCC	FP	CBLC
32	TAGAAAGTCCCCAGAGCCGCCG	RP	
33	GCTTCGACCAGTTCTACGAGGCAGC	FP	HOXD11
34	CGCTTGCCCAGTACCCGGTGCC	RP	
35	AGTCAATCGTGGGCTGCATCATC	FP	KCNJ2
36	GGGGACACCAGAAATATACGATCG	RP	
37	TAGGGCTCTGACCTTGTTCCAG	FP	ZSCAN18
38	CTGCTGGATGACGGACTGCCT	RP	
39	CCATGCTCCGCCCCTCTAG	FP	KCTD17
40	ACAGGCTGAGGCGGGTGGG	RP	
41	GATGATCAGTACCAAGGCTGTAGC	FP	ART4
42	AGAGGAATTGGCCAAATCGAATGG	RP	
43	GCCCTCCTGCTCTGTCCTCCAG	FP	BPIFB5
44	CCAGCCACAGAGCCCCTCAC	RP	
45	GGCTTTCACCATCTCTATGCTTTTGGG	FP	AVPR1B
46	TTAGGCTGAGGCTGAGGCTGAG	RP	
47	CGCCAGTGCGGTGTCTCTG	FP	ATAD3A
48	CTGGATGGGGTGCCGCAGCG	RP	
49	CCTTGTTCTCTGTGCCCACAC	FP	ALDH3B2
50	TCACAGGAGGGTGCAGCTCTG	RP	
51	GTCAGGTTGCTAAGCAGTGAAAG	FP	EME1
52	GGGCAGGTAGACAGGTAGGC	RP	
53	CGGGGGAAGTACAGCGACC	FP	CHST11
54	GGTGGCGTTCTTCCGCTGGCG	RP	
55	ACTCCGGTCCCCCCTCCTCCT	FP	MMP9
56	GGCGAGCCCCCTCACTCAC	RP	
57	CTGGAAACTCCTCTGCCTCC	FP	UROC1
58	GGTGGCAAGGACTGGGCTGAC	RP	
59	CAACCTGACTTTCTTCTTCTCC	FP	SFTPD
60	GCCCTCTGTCTTGGAATCAGTC	RP	
61	CTCTCCTTTTTGTCTTCATCCAC	FP	KCNH8
62	GTGCTCCGAGTTGTGGACAG	RP	
63	GCTGAGCGCATGGCCATCCGGCA	FP	B3GALT5
64	TCATGGTCTTCAGGGTCAGATTG	RP	
65	GTCCCCAGGAGCACCGTGAG	FP	TBX5
66	GACCAGGGGTGATCACACTCAC	RP	
67	GCTCACCTCATTGAACACCAGAGAG	FP	ZNF419
68	GGTCTTTCTCCAGTGTGAATTCTC	RP	
69	CTCACTCTGTTGCCTCTACCCC	FP	BPI
70	CACCCACCCAGAGCCCGGC	RP	
71	GGAGTGGCTGAATGCTTCCTC	FP	OR10A2
72	CACAGACCAGCCTCAGCACAG	RP	
73	TGGCTCTTATTCTTCCGGCTTC	FP	NRP2
74	GAAGAGACAGATATTCACATACCC	RP	
75	TGATCTTTGCTCTCCCCCTG	FP	ALPPL2
76	CTGGCTCACTCACCGTCACCC	RP	
77	CAGAGTTCCCACCTCCCTCCC	FP	CYP4F2
78	TCAGCTCAGGGGCTCCACCCGC	RP	
79	CATGATCTCTGGGGTTTGTTTCC	FP	DAPK1
80	GGGATCCTGTTCTCTGTGCATAC	RP	
81	GAGAAGGAAAATTATGACGTTTGGGC	FP	ARHGAP25
82	TTAAGCCTCGGTCTTGGGTTCCTTC	RP	
83	ACGCCCAGTCTTCCCCCCCC	FP	H6PD
84	TGCGCAGGGCCACTCACACAC	RP	
85	TTGCCCAAATATCACAGTGTGTA	FP	TEP1
86	CCCGGCCTAGAACCCAACCT	RP	
87	CTCTTCTTCAGTTACAAGTTCCCCC	FP	ACSM2B
88	CCATGGTTCCCCTGGGAACAGG	RP	
89	GGATCTGTGATGAGAACGGAGCC	FP	VWF
90	CAGTGGTAAGAGGAGGACCTGGC	RP	
91	AATCTCGCTCTTCTCTTACCTC	FP	ABCA4
92	GCATAAGCAGCAGGGGTACCTGG	RP	
93	GTTTTTAGCTCCCTGTGCTGTC	FP	PCMTD1
94	CTGTCTAGAGGCTGAGGAATAAGCTG	RP	
95	GGCACGGGGGTGTGAACCAGC	FP	PAX2
96	CCGGGACAGCTGCGAAGCCCTC	RP	
97	CTTTCACCTCGGGCAAATATTACTGG	FP	TRIM49
98	CACAGTCTTAGCCTCACAATCCAGG	RP	
99	GAATGTAAGGAATGTGGGAAAACC	FP	ZNF717
100	CTTCTTGTTGGTAGGCCAGGAGG	RP	
101	GAGGTGGCCAACGGGCTGAGGAAC	FP	HHIPL2
102	CTTGCGATTGTGGCGGAATTTGGG	RP	
103	CGCTCAACAGAACAGAGGTGTCCG	FP	OR13J1
104	GACAGGAGGTGGACCAGCATCAGAG	RP	
105	GTCCATCTGGGAAGCGGGATTTGG	FP	BEND5
106	GTGACCTACCTCTGACGATGCTTAGTTTG	RP	
107	TTCCCCAGCCCTTCGAGATAATCG	FP	CADM3
108	GACACTGGGGTTTGGGAGTCTCAC	RP	
109	CAGCTGGTCTGTCAATGTCTGTGGG	FP	JMJD4
110	GGGGGCAGGAGAAGCAGCACATTAC	RP	
111	TACAGATTCCTCAGTGTCTTATATTG	FP	CDC27
112	GTATCTTGTTTGACTTACCTTGGGG	RP	
113	CCAGCCCCTCCCGCCTCCTGCA	FP	CAPN5
114	CCTCAAGCCAGCCCCCAAGTAC	RP	
115	GTCATGTTTCTTTTTTTCTCTAGAGT	FP	SCAF8
116	CTGGAGTATTGCTCATAGCAGTGG	RP	
117	CTTACTCTTCTGCCCTCTTCTTCAG	FP	PGLS
118	GGGCCGCGTGCCTGGTCCCAG	RP	
119	GCCAAGCGGCGTGCGGCCGAG	FP	C9ORF40
120	CGATGCGACCCCCCAGTCTCCCC	RP	
121	ATGTCCCGGCACCATAGCCGCTTC	FP	KLHL29
122	TGGTGACCGCAGACGCAGAGCTGGC	RP	
123	GCTCAAGCCCCTCCTTCCCTT	FP	PFDN5
124	TGGTCCAGCTGGTTCTTGAGCATTTC	RP	
125	AAGATCTCCCAAAGGCTGTGGTG	FP	FCGR3A
126	TTCCTCTTCCCCTTCATCAACTC	RP	
127	TCCCTCCCACCCTTGTCCTGCAG	FP	HYDIN
128	GCTGTCCAATGTGAGTGGTCAGTAC	RP	
129	GGGCCGTTGGTGGGAGCGGTG	FP	GK2
130	CTTGTCCCAGATTACAGTGGTTTCC	RP	
131	CAAGTGGAGAAGTCTGTCAACGATGC	FP	ZNF578
132	CTACAATTAAAGGCTTCGCCAGTCTC	RP	
133	CCAAGGAAGACCCCAAAGCACTG	FP	DFFA
134	CTGGCTCGCTTAGGATTCTGCAGGTC	RP	
135	TGGCCTCGTCCTGGGCTCCGA	FP	CHRNA1
136	GGCCCCCAGTGCTCTTGTCTCAC	RP	
137	GCCCGCAACCCCGTCCCTGAAGGTG	FP	TRIM15
138	AGTCTCTGCCTGCTCCTCCTCTTGGC	RP	
139	CACCCCATGCTATTGTTTATCC	FP	PNPLA1
140	CCTGCCGTGCTCGAAGGCTTG	RP	
141	CACTCATTCTCTTGTGGGG	FP	CLTB
142	CCTCATGTCCCTACAGCCCTC	RP	
143	CAGCTTGGTCTCTGGGAACAC	FP	GRIN2A
144	CCTCTCCATCTGCCCGTAGCAG	RP	
145	ATCCCCTCTGGCACGGCCCGCA	FP	GIPC3
146	CAGGGGGCTGGGCTCCGGGCTG	RP	
147	AGGAAGCACCAGTTCAAGATCAAG	FP	ZNF8
148	GTCACTGTTTTCACCGGGCTGTTTTCC	RP	
149	CACTTCTGGGCGTGCCCTGCAG	FP	ANKRD24
150	TCACTCCCGGGTCGCGGGCACTC	RP	
151	CACATTTCATTTCCTTTGTTTTCAGAC	FP	C10ORF67
152	CAGAACAAATTCAGATTTACCTGTGC	RP	
153	CAGCTACAGATTAGCACCTAAGTATC	FP	AADAC
154	ATGAACATACCTGTTGAGTCACTGC	RP	

Supplementary Table 5

List of genetic variants and primer sequences used for Sanger sequencing
